# South Asian ethnicity and material deprivation increase the risk of Epstein-Barr virus infection in childhood Hodgkin's disease

**DOI:** 10.1054/bjoc.2001.1872

**Published:** 2001-08

**Authors:** K J Flavell, J P Biddulph, J E Powell, S E Parkes, D Redfern, M Weinreb, P Nelson, J R Mann, L S Young, P G Murray

**Affiliations:** School of Health Sciences, University of Wolverhampton, Wolverhampton, WV1 1DJ; Department of Epidemiology and Public Health, UCL (University College London), 1–19 Torrington Place, London, WC1E 6BT; Department of Public Health & Epidemiology, University of Birmingham, Edgbaston, Birmingham, B15 2TT; Department of Oncology, The Children’s Hospital, Steelhouse Lane, Birmingham, B4 6NH; Department of Pathology, The Children’s Hospital, Steelhouse Lane, Birmingham, B4 6NH; Division of Cancer Studies, University of Birmingham, Edgbaston, Birmingham, B15 2TT, UK

**Keywords:** Hodgkin's disease, Epstein-Barr virus, ethnicity, childhood, material deprivation

## Abstract

In order to further define the factors associated with the observed variations in the Epstein-Barr virus-positive rate in childhood Hodgkin's disease, we have studied the effect of material deprivation (measured by the Townsend score) and ethnic origin on the frequency of Epstein-Barr virus-positivity in 55 cases of childhood Hodgkin's disease, diagnosed between 1981 and 1999, from a multi-ethnic region of the United Kingdom. Epstein-Barr virus status was determined by immunohistochemistry for the Epstein-Barr virus-encoded latent membrane protein-1. 62% of cases were Epstein-Barr virus-positive. Ethnic group was the strongest predictor of Epstein-Barr virus-positivity, with South Asians having a more than 20-fold risk of being Epstein-Barr virus-positive compared with non-South Asians. An increased risk was still present after adjusting for deprivation. Townsend scores were significantly higher (indicating more deprivation) in the Epstein-Barr virus-positive group, particularly in males. The relative risk of Epstein-Barr virus-positivity showed a gradient with increasing Townsend score; the risk being 7-times higher in the most deprived quartile compared with the least deprived group. Although the association between Townsend score and Epstein-Barr virus-positivity was reduced after adjusting for ethnic group, the risk of Epstein-Barr virus-positivity was still 3-times higher in the most deprived compared with the least deprived quartile. In addition, cases having 2 or more siblings were 5-times as likely to be Epstein-Barr virus-positive as those from smaller families. These results provide the first evidence of a strong association between Epstein-Barr virus-positive Hodgkin's disease and South Asian children from the United Kingdom. In addition, deprivation may increase the likelihood of Epstein-Barr virus-positive disease independently of ethnicity. © 2001 Cancer Research Campaign http://www.bjcancer.com


*British Journal of Cancer* (2001) **85**(3), 350–356
© 2001 Cancer Research Campaign
doi: 10.1054/ bjoc.2001.1872, available online at http://www.idealibrary.com on IDEAL®

http://www.bjcancer.com

### South Asian ethnicity and material deprivation increase the risk of Epstein-Barr virus infection in childhood Hodgkin's disease

**KJ Flavell[1], JP Biddulph[2], JE Powell[3], SE Parkes[4], D Redfern[5], M Weinreb[4], P Nelson[1], JR Mann[4], LS Young[6] and PG Murray[6]**

[1]School of Health Sciences, University of Wolverhampton, Wolverhampton, WV1 1DJ; [2]Department of Epidemiology and Public Health, UCL (University College London), 1–19 Torrington Place, London, WC1E 6BT; [3]Department of Public Health & Epidemiology, University of Birmingham, Edgbaston, Birmingham, B15 2TT; [4]Department of Oncology, The Children's Hospital, Steelhouse Lane, Birmingham, B4 6NH; [5]Department of Pathology, The Children's Hospital, Steelhouse Lane, Birmingham, B4 6NH; [6]Division of Cancer Studies, University of Birmingham, Edgbaston, Birmingham, B15 2TT, UK

**Summary** In order to further define the factors associated with the observed variations in the Epstein-Barr virus-positive rate in childhood Hodgkin's disease, we have studied the effect of material deprivation (measured by the Townsend score) and ethnic origin on the frequency of Epstein-Barr virus-positivity in 55 cases of childhood Hodgkin's disease, diagnosed between 1981 and 1999, from a multi-ethnic region of the United Kingdom. Epstein-Barr virus status was determined by immunohistochemistry for the Epstein-Barr virus-encoded latent membrane protein-1. 62% of cases were Epstein-Barr virus-positive. Ethnic group was the strongest predictor of Epstein-Barr virus-positivity, with South Asians having a more than 20-fold risk of being Epstein-Barr virus-positive compared with non-South Asians. An increased risk was still present after adjusting for deprivation. Townsend scores were significantly higher (indicating more deprivation) in the Epstein-Barr virus-positive group, particularly in males. The relative risk of Epstein-Barr virus-positivity showed a gradient with increasing Townsend score; the risk being 7-times higher in the most deprived quartile compared with the least deprived group. Although the association between Townsend score and Epstein-Barr virus-positivity was reduced after adjusting for ethnic group, the risk of Epstein-Barr virus-positivity was still 3-times higher in the most deprived compared with the least deprived quartile. In addition, cases having 2 or more siblings were 5-times as likely to be Epstein-Barr virus-positive as those from smaller families. These results provide the first evidence of a strong association between Epstein-Barr virus-positive Hodgkin's disease and South Asian children from the United Kingdom. In addition, deprivation may increase the likelihood of Epstein-Barr virus-positive disease independently of ethnicity. © 2001 Cancer Research Campaign http://www.bjcancer.com

**Keywords**: Hodgkin's disease; Epstein-Barr virus; ethnicity; childhood; material deprivation

The age incidence of Hodgkin's disease (HD) is bimodal. Developed countries are characterized by an initial peak in early adulthood, whereas in developing countries this peak occurs in childhood. This has led some to suggest a possible infectious aetiology for those cases of HD that account for the first peak in the incidence, with infection occurring earlier in individuals from less privileged communities (Gutensohn and Cole, 1980; Alexander et al, 1991).

Substantial evidence has now accumulated to link the Epstein-Barr virus (EBV) with the pathogenesis of HD. Evidence for this association at the cellular level includes the localization of EBV DNA (Weiss et al, 1989) and the latent gene products, which include the EBV-encoded early RNAs (Wu et al, 1990), Epstein-Barr nuclear antigen-1 (Grässer et al, 1994), latent membrane protein-1 (LMP1) (Pallesen et al, 1991) and LMP2 (Niedobitek et al, 1997), to the malignant Hodgkin and Reed-Sternberg (HRS) cells of HD.

The frequency of EBV-associated HD (EBV-positive rate) has been found to vary with age, sex, cellular subtype, ethnicity and country of residence (Glaser et al, 1997). For country of residence, the EBV-positive rate appears to vary with the level of socioeconomic development of the country, being lower in developed countries compared with developing countries. In childhood HD, variation of the EBV-positive rate is also apparent (Glaser et al, 1997). However, international differences in EBV-positivity may not be attributable solely to environmental factors such as deprivation, since substantial genetic variation also exists between the populations studied. Therefore, studies based within a smaller region of a single country may be more informative with regard to the specific factors responsible for variation in the EBV-positive rate within HD.

In an earlier study of predominantly adult cases within a multi-ethnic region of the United Kingdom (Flavell et al, 1999), we were able to show that EBV-positive HD was associated with a higher level of relative deprivation compared with EBV-negative HD cases, although we were unable to adjust for an individual's ethnic origin. The aim of the present study therefore, was to examine the effect of material deprivation and ethnic group on the EBV-positive rate in childhood HD from a single area of the United Kingdom. Material deprivation was measured in terms of Townsend score and the individual components of the Townsend score were also analysed separately to investigate if any of these were associated with the risk of EBV-positivity. This should

*Received 25 October 2000*
*Revised 21 March 2001*
*Accepted 4 April 2001*

*Correspondence to:* PG Murray enable further definition of the factors within such populations that could be responsible for the variation in the EBV-positive rate in childhood HD.

### MATERIALS AND METHODS

### Study area

The study area was the West Midlands National Health Service (NHS) Region, which according to the Office of National Statistics at the 1991 census contained 1 003 754 children under 15 years, 98 177 (9.8%) of whom were 'South Asian' (i.e. ethnic groups 'Indian', 'Pakistani' or 'Bangladeshi'). South Asians comprise the largest single ethnic minority group in the United Kingdom.

### Patient characteristics

All cases of HD in children under 15 years of age at diagnosis, residing in the study area between 1981 and 1999, were eligible for inclusion in the study. The case series consisted of eligible cases, identified by the West Midlands Regional Children's Tumour Registry (WMRCTR), for whom archival pathological material (paraffin wax-embedded tissue blocks) were available. The WMRCTR is a population-based registry that routinely reviews the pathology of HD and other tumours. Patient data (date of birth, date of diagnosis, sex, ethnic group, histological subtype, disease stage, social class, birth order, number of siblings and residential postcode at diagnosis) were extracted from medical notes by the WMRCTR. Ethnic group was recorded as one of 3 categories; 'White', 'South Asian' (ethnic groups 'Indian', 'Pakistani' or 'Bangladeshi') and 'Other' (including 'Black', 'Oriental' and 'mixed race'). Histological subtype was classified according to the Rye system (Lukes et al, 1966) and based upon consensus morphology as determined by the WMRCTR.

The social class of the patient was derived from the occupation of the main income earner of the family, coded using the Standard Occupational Classification (OPCS, 1991). The effect of the patient's birth order and the number of siblings at diagnosis were also investigated, as these factors, along with overcrowding are thought to affect patterns of childhood infection (Gutensohn and Cole, 1981).

For quantifying material deprivation, a 1991 census-based deprivation measure, the Townsend score, was employed. This score is derived from the Enumeration District (the smallest unit of population for which data were available) in which the patient resided at diagnosis, as determined from the patient's postcode. The Townsend score is a widely accepted indicator of material deprivation (Morris and Carstairs, 1991) and is calculated from the following 4 variables (Townsend et al, 1988).

1. Percentage of economically active residents (aged 16–64 for men, 16–59 for women) who are unemployed;
2. Percentage of households without a car;
3. Percentage of households not owner-occupied;
4. Percentage of households that are overcrowded (more than one person per room).

The individual components of the Townsend score were also obtained for each Enumeration District.

To determine whether the case series was a representative sample of all childhood HD cases diagnosed in the West Midlands NHS Region between 1981 and 1999, we compared the patient characteristics of the case series with those of the cases for whom no pathological material was available. Within the West Midlands NHS Region, the residence of the patients was categorized as West Midlands county or surrounding shires. The West Midlands county residents being nearer to the Region's paediatric oncology centre and the base of the WMRCTR.

### Detection of latent EBV infection

4 μm paraffin sections were prepared, deparaffinized and washed in phosphate-buffered saline pH 7.6. Immunohistochemistry for LMP1 was performed to detect the presence of EBV infection in the malignant HRS cells of HD. The Biogenex StrAviGen Multilink kit (Biogenex Ltd. Cat no. LP000-U-L) was employed using the primary monoclonal antibody reagent, CS1–4, diluted 1:25 in phosphate-buffered saline (Murray et al, 1996). Prior to immunostaining, sections were pre-treated by pressure cooking for 2 minutes in 0.01 M citrate buffer pH 6.0. Positive controls for LMP1 consisted of known LMP1-expressing tumours, including HD tissues, processed in the same way as test samples. Negative controls consisted of consecutive test sections in which primary antibody was replaced with buffer. Tumour specimens were recorded as either EBV-positive (LMP1 present within HRS cells) or EBV-negative (LMP1 not detectable in HRS cells).

### Statistical methods

The Mann–Whitney U test was used to detect differences between EBV-positive and EBV-negative groups for Townsend score. Chi-squared test with Yates' continuity correction (1 df) (or 2-sided Fisher's exact test where numbers were small) was used to evaluate differences in the distribution of patient and disease characteristics between the EBV-positive and EBV-negative groups. Logistic regression analysis was used to determine which factors were independently associated with EBV-positive status. Analyses were repeated excluding unclassifiable cases and those of the lymphocyte predominant (LP) subtype as LP disease is believed to be an entity distinct from classic HD (Harris et al, 1994). All analyses used SPSS Version 9 (SPSS Inc., Chicago, USA) and differences were deemed significant if the $P$ value was less than 0.05.

### RESULTS

Between 1981 and 1999, 113 cases of HD were diagnosed in children under 15 years old who were resident in the West Midlands NHS Region. Of these eligible cases, pathological material from 58 cases were available from the WMRCTR.

### Representativeness of the case series

The distribution of patient characteristics for cases that were and were not included in our case series is shown in Table 1. The case series was more likely to contain recently diagnosed cases ($P$ = 0.006) and those who were diagnosed and treated at the Region's paediatric oncology centre ($P$ = 0.002) in Birmingham. Ethnic minority groups, most of whom reside in the West Midlands county rather than in the surrounding shires, were also over-represented ($P$ = 0.001). However, no significant differences were found in age, sex, disease stage or histological subtype.

© 2001 Cancer Research Campaign

**Table 1** Characteristics of 113 cases of childhood HD from the West Midlands NHS Region, 1981–1999

| | | Number (%) | | |
|---|---|---|---|---|
| | | **Material available** | **Material not available** | ***P*[a]** |
| | Total cases | 58 | 55 | |
| Age | | | | 0.326 |
| | 0–9 years | 23 (40) | 16 (29) | |
| | 10–14 years | 35 (60) | 39 (71) | |
| Sex | | | | 0.689 |
| | Male | 40 (69) | 35 (64) | |
| | Female | 18 (31) | 20 (36) | |
| Ethnic group | | | | 0.001 |
| | White | 36 (62) | 43 (78) | |
| | South Asian | 20 (35) | 5 (9) | |
| | Other | 2 (3) | 7 (13) | |
| Stage at diagnosis | | | | 0.993 |
| | I & II | 40 (69) | 37 (67) | |
| | III & IV | 18 (31) | 18 (33) | |
| Disease subtype[b] | | | | 0.558 |
| | LP | 14 (24) | 17 (31) | |
| | MC | 9 (16) | 11 (20) | |
| | NS | 32 (55) | 26 (47) | |
| | U | 3 (5) | 1 (2) | |
| Year of diagnosis | | | | 0.006 |
| | 1981–90 | 21 (36) | 35 (64) | |
| | 1991–99 | 37 (64) | 20 (36) | |
| Diagnosed at | | | | 0.002 |
| | Local hospital | 4 (7) | 17 (31) | |
| | Regional centre | 54 (93) | 38 (69) | |
| Residence | | | | 0.110 |
| | West Midlands County | 34 (59) | 23 (42) | |
| | Surrounding Shires | 24 (41) | 32 (58) | |

[a]Chi-squared test (with Yates' correction in 2×2 table) Fisher's exact test was used where cell counts were small.
[b]LP = lymphocyte predominant; MC = mixed cellularity; NS = nodular sclerosis; U = unclassifiable.

### EBV status and patient characteristics

EBV status was unequivocally determined for 55 of the 58 (95%) samples examined (3 cases were excluded where contradictory initial results could not be resolved due to a lack of pathological material). EBV-positivity was found in 62% (34/55) of cases (Table 2). EBV-positive status was strongly associated with ethnic group (95%; 18/19 of South Asians were positive compared with 44%; 16/36 of non-South Asians, $P < 0.001$). Males were also more likely than females to be EBV-positive, though this did not reach significance (70%; 26/37 compared with 44%; 8/18 respectively, $P = 0.081$). EBV status was unaffected by age and disease stage. Of the classifiable subtypes mixed cellularity (MC) had the highest percentage of EBV-positive tumours at 75% (6/8), compared with 66% (21/32) of nodular sclerosis (NS) and 38% (5/13) of LP, but these differences were not significant ($P = 0.186$).

### EBV status, ethnicity and material deprivation

The Townsend scores for the case series ranged from –4.9 to +7.8 (median +1.1, interquartile range –1.8 to +3.9). The EBV-positive group was associated with higher relative deprivation scores (median score of +3.2) compared with the EBV-negative group (median score of –0.4) and this difference between groups was significant ($P = 0.015$, Table 3). The same pattern was observed when the sexes were examined separately (males EBV-positive median score +3.0, EBV-negative –0.8, $P = 0.009$; females +4.0, +0.4, $P = 0.657$), though the difference was significant only for the males. Similarly, when the series was grouped by age-band, the Townsend scores were higher in the EBV-positive than in the EBV-negative patients in both age categories (0–9 years +3.5, –1.8, $P = 0.052$: 10–14 years +3.2, –0.4, $P = 0.167$) and was of borderline significance for the 0–9 year olds.

After grouping the sample into 4 quartiles (using SPSS's categorization routine) based on increasing Townsend score, a significant trend ($P = 0.025$) was observed (Table 2), with EBV-positive cases being more common in the more deprived quartiles. However, no significant association was found between EBV status and social class. EBV-positivity was not associated with birth order, but children having 2 or more siblings were significantly more likely to be EBV-positive (74%; 23/31 compared with 37%; 7/19, $P = 0.016$).

Logistic regression was then used to derive odds ratio estimates of the relative risk of EBV-positivity for each individual factor (Table 4). Ethnic group was the single factor most strongly associated with EBV-positivity, with South Asians having a more than 20-fold risk of being EBV-positive compared with non-South Asians. While the relative risk of EBV-positivity showed a gradient with increasing Townsend score, there was a notable rise for the most deprived quartile, where the risk was 7-times as high as in the least deprived group. Cases having 2 or more siblings were 5-times as likely to be EBV-positive as those from smaller families.

However, after adjusting for the effects of ethnic group, the only factor independently associated with EBV-positivity was sex, with

© 2001 Cancer Research Campaign

**Table 2** EBV status of 55[a] cases of childhood HD from the West Midlands

| | | No. | No. (%) EBV-positive | Association with EBV-positivity (*P*)[b] |
|---|---|---|---|---|
| | Total cases | 55 | 34 (62) | |
| Age | | | | 0.779 |
| | 0–9 years | 20 | 13 (65) | |
| | 10–14 years | 35 | 21 (60) | |
| Sex | | | | 0.081 |
| | Male | 37 | 26 (70) | |
| | Female | 18 | 8 (44) | |
| Ethnic group | | | | <0.001 |
| | South Asian | 19 | 18 (95) | |
| | White and Other | 36 | 16 (44) | |
| Stage at diagnosis | | | | 0.386 |
| | I & II | 38 | 25 (66) | |
| | III & IV | 17 | 9 (53) | |
| Disease subtype | | | | 0.186 |
| | LP | 13 | 5 (38) | |
| | MC | 8 | 6 (75) | |
| | NS | 32 | 21 (66) | |
| | U | 2 | 2 (100) | |
| Townsend score | | | | 0.025[c] |
| | Quartile 1 (least deprived) | 13 | 6 (46) | |
| | Quartile 2 | 14 | 7 (50) | |
| | Quartile 3 | 14 | 9 (64) | |
| | Quartile 4 (most deprived) | 14 | 12 (86) | |
| Social class[d] | | | | 0.363 |
| | Non-manual occupations | 16 | 8 (50) | |
| | Manual occupations | 38 | 25 (66) | |
| Birth order[e] | | | | 1.000 |
| | First born child | 20 | 12 (60) | |
| | Not first born | 30 | 18 (60) | |
| No. siblings[e] | | | | 0.016 |
| | 1 or fewer | 19 | 7 (37) | |
| | 2 or more | 31 | 23 (74) | |

[a]Excluding 3 cases where EBV test was not informative; [b]Fisher's exact test; [c]test for trend; [d]excluding 1 case where social class was not known; [e]excluding 5 cases where birth details were not known.

males having more than 4-times the risk of EBV-positivity as females. A high Townsend score was no longer significantly associated with EBV-positivity, though the odds ratio was 3.1 (95% CI 0.4–24.2) for the most deprived quartile. The magnitude of the ethnic effect was similar in all models, the point estimate of the odds ratio ranging from 17.1 to 30.8 and the lower 95% confidence limit of the odds ratio never falling below 2.0, indicating at least a doubled risk of EBV-positivity in South Asians.

The analysis also adjusted for Townsend score (as a continuous variable). Ethnic group was independently associated with EBV-positivity with an odds ratio of 17.1 (95% CI 2.0–146.7) indicating that irrespective of the level of deprivation, ethnicity is still a risk factor for EBV-positivity. The number of siblings was also a risk factor independent of deprivation, although not when ethnicity was controlled for.

A logistic regression analysis was performed using the individual components of the Townsend score, as continuous variables. Ethnic group was still the strongest predictor of EBV status, followed by sex. When odds ratios for the 4 individual components of the Townsend score were calculated, the only significant risk factor was unemployment (Table 4) but this was no longer significant when ethnicity was controlled for.

**Table 3** Townsend scores for 55[a] cases by age and sex

| | | EBV-positive | | | | EBV-negative | | | | |
|---|---|---|---|---|---|---|---|---|---|---|
| | No. of patients | No. (%) | 25th Centile | Median | 75th Centile | No. | 25th Centile | Median | 75th Centile | Mann–Whitney test (*P*) |
| All patients | 55 | 34 (62) | –0.59 | +3.24 | +5.00 | 21 | –3.02 | –0.42 | +1.67 | 0.015 |
| All male patients | 37 | 26 (70) | –0.36 | +2.97 | +5.34 | 11 | –3.61 | –0.81 | +1.11 | 0.009 |
| All female patients | 18 | 8 (44) | –3.04 | +4.04 | +4.85 | 10 | –2.36 | +0.38 | +3.78 | 0.657 |
| All patients 0–9 years | 20 | 13 (65) | –0.46 | +3.48 | +5.31 | 7 | –3.81 | –1.85 | +1.11 | 0.052 |
| All patients 10–14 years | 35 | 21 (60) | –0.59 | +3.23 | +4.97 | 14 | –2.04 | –0.40 | +3.47 | 0.167 |

[a]Excluding 3 cases where EBV test was not informative.

*© 2001 Cancer Research Campaign* 

**Table 4** Association between EBV-positivity and patient characteristics: logistic regression analysis

| | 'Relative risk' of EBV-positive status | | | | | |
|---|---|---|---|---|---|---|
| | | | Odds ratio (OR) adjusted for | | | |
| | Crude OR | 95% CI | Ethnic group | 95% CI | TS | 95% CI |
| Ethnic group | | | | | | |
| Non-South Asian | 1.00 | – | – | – | 1.00 | – |
| South Asian | 22.47 | 2.71–187 | – | – | 17.13 | 2.00–147 |
| Age | | | | | | |
| Per year of age increase | 0.91 | 0.76–1.08 | 0.83 | 0.68–1.02 | 0.89 | 0.73–1.07 |
| 0–9 years | 1.00 | – | 1.00 | – | 1.00 | – |
| 10–14 years | 0.81 | 0.26–2.53 | 0.50 | 0.14–1.85 | 0.69 | 0.20–2.37 |
| Sex | | | | | | |
| Female | 1.00 | – | 1.00 | – | 1.00 | – |
| Male | 2.95 | 0.92–9.49 | 4.42 | 1.003–19.5 | 3.25 | 0.92–11.5 |
| Disease stage | | | | | | |
| I & II | 1.00 | – | 1.00 | – | 1.00 | – |
| III & IV | 0.59 | 0.18–1.87 | 0.61 | 0.16–2.34 | 0.53 | 0.15–1.85 |
| Subtype | | | | | | |
| LP | 1.00 | – | 1.00 | – | 1.00 | – |
| NS + MC | 3.32 | 0.91–12.2 | 3.28 | 0.70–15.3 | 2.49 | 0.63–9.81 |
| Townsend score (TS) | | | | | | |
| per unit increase | 1.25 | 1.04–1.49 | 1.16 | 0.95–1.41 | – | – |
| Quartile 1 (least deprived) | 1.00 | – | 1.00 | – | – | – |
| Quartile 2 | 1.17 | 0.26–5.29 | 1.03 | 0.20–5.42 | – | – |
| Quartile 3 | 2.10 | 0.45–9.84 | 1.50 | 0.27–8.29 | – | – |
| Quartile 4 (most deprived) | 7.00 | 1.10–44.6 | 3.09 | 0.40–24.2 | – | – |
| TS components | | | | | | |
| per unit increase | | | | | | |
| Unemployment | 1.11 | 1.03–1.20 | 1.08 | 0.99–1.17 | – | – |
| Overcrowding | 1.16 | 0.99–1.36 | 1.01 | 0.85–1.21 | – | – |
| Car ownership | 1.04 | 1.00–1.07 | 1.03 | 0.99–1.06 | – | – |
| Owner occupied | 1.02 | 0.99–1.04 | 1.02 | 1.00–1.05 | – | – |
| Social class | | | | | | |
| Non-manual occupations | 1.00 | – | 1.00 | – | 1.00 | – |
| Manual occupations | 1.92 | 0.59–6.30 | 1.69 | 0.44–6.54 | 0.83 | 0.20–3.44 |
| Birth order | | | | | | |
| First born child | 1.00 | – | 1.00 | | 1.00 | – |
| Not first born | 1.00 | 0.32–3.17 | 1.65 | 0.39–6.89 | 1.07 | 0.31–3.65 |
| No. siblings | | | | | | |
| 1 or fewer | 1.00 | – | 1.00 | – | 1.00 | – |
| 2 or more | 4.93 | 1.44–16.9 | 3.13 | 0.77–12.6 | 3.73 | 1.02–13.6 |

After exclusion of LP and unclassifiable subtypes, 40 cases remained. Higher relative deprivation scores were observed for EBV-positive compared with EBV-negative patients from this subgroup (median scores of +3.3 and +0.3 respectively) but this difference was not significant ($P = 0.091$). On univariate analysis of these patients the only factors significantly associated with EBV-positivity were ethnic group (odds ratio 12.9; 95% CI 1.5–113.7) and the unemployment measure (odds ratio 1.1; 95% CI 1.0–1.2). After adjusting for ethnic group, age (in years) was the only factor independently associated with EBV-positivity (odds ratio 0.8; 95% CI 0.6–1.0). After adjusting for Townsend score, ethnic group was the only factor independently associated with EBV-positivity (odds ratio 10.9; 95% CI 1.2–98.6).

### DISCUSSION

International differences in the EBV-positive rate in childhood HD may suggest a role for socioeconomic factors in the aetiology of EBV-positive childhood HD (Glaser et al, 1997). However, numerous other factors vary between countries and adjustment for some of these variables reduces the apparent impact of geographical differences (Armstrong et al, 1993; Razzouk et al, 1997). Through the use of a population-based study of a region within a single country, we have been able to control for many of these variables. Since some ethnic minority groups in the United Kingdom are known to be more materially disadvantaged than other ethnic groups, adjustment for ethnic group is necessary when studying deprivation.

The most important factor found to affect EBV status in our analysis was ethnic group, even after adjusting for material deprivation. Ethnic differences were reported in a Malaysian study where Indians were found to have the highest percentage of EBV-positive cases compared with Chinese and Malay patients (Peh et al, 1997). Ethnic or racial variation in EBV-positive rates in HD have also been demonstrated in the USA, where EBV-positive cases were found to be more common in Hispanic American chil-

 *© 2001 Cancer Research Campaign*

dren than in black or white American children with HD (Ambinder et al, 1993). While several earlier studies in the United Kingdom have indicated that South Asian children, particularly those aged 0–9 years, have a higher risk of developing HD than their white counterparts (Stiller et al, 1991; Powell et al, 1994), EBV-positive rates were not examined.

Only one study has examined the effects of both ethnic group and deprivation (indicated by the per capita gross national product) on the frequency of EBV-positivity in childhood HD (Glaser et al, 1997) and this international comparison found that the 2 factors were independent predictors of the risk of EBV-positive Hodgkin's disease. We have shown significantly higher levels of relative deprivation (higher Townsend scores) in the EBV-positive childhood HD cases compared with EBV-negative cases. This was similar to our previous results on a series of predominantly adult patients (Flavell et al, 1999) from which only 7 children were included in the present study. While this association was no longer significant after adjusting for ethnic group, the most deprived quartile (Townsend score greater than +3.7) still showed a 3-fold relative risk of EBV-positivity compared with the least deprived quartile. This suggests that within a single geographical region, socioeconomic factors could contribute independently of ethnicity to the association of EBV-infection with childhood HD.

We found no significant association between EBV status and social class. While social class (derived from individual data) might be expected to reflect deprivation more accurately than the Townsend score (a group measurement), there is a need to consider which specific aspects of deprivation are most likely to influence EBV status. We observed a 5-fold increased risk of EBV-positivity among children having 2 or more siblings, though this effect was reduced after adjusting for ethnicity and deprivation. The Townsend score contains a measure of 'overcrowding', and in developed countries where parasitic disease and malnutrition are rare, overcrowding may be the specific aspect of deprivation that is most closely linked to EBV infection. However, when the individual components of the Townsend score were analysed by univariate analysis, unemployment was the only significant risk factor. When interpreting these results one must consider that the Townsend score (and its individual components) is based on a single year's census data and must necessarily become less accurate as one moves away from the census year.

The EBV-positive rate in our study was higher in males than females, as has been shown by others (Preciado et al, 1995; Andriko et al, 1997). In addition, both male and female children with EBV-positive HD had higher median Townsend scores than their EBV-negative counterparts. This difference was significant in the males. The association between deprivation and EBV-positivity was stronger in children under 10 years of age, than those aged 10–14 years, although the EBV-positive rates were similar (Table 3) in each age group. Conflicting data on EBV-positive rates with age exist, with some observing higher EBV-positive rates in younger children (Jarrett et al, 1996; Armstrong et al, 1998), whilst others have shown similar EBV-positive rates for each childhood age group (Weinreb et al, 1992). These differences could be explained by small numbers in some of the cases series (Armstrong et al, 1998) or may reflect differences in the composition of cases between series.

It has been suggested that the LP subtype is usually an EBV-negative disease (Harris et al, 1994). We identified 38% of LP cases as EBV-positive. Inconsistencies in the classification of LP by morphology have been identified (von Wasielewski et al, 1997) and may account for these discrepancies. LP usually has an immunophenotype distinct from the other classic subtypes of HD (Harris et al, 1994). Due to the lack of availability of tissue sections, we were not able to produce complete immunophenotypic profiles from all LP cases and we therefore performed statistical analyses with and without the LP and unclassifiable cases. The overall trends were the same for both groups and ethnicity remained the most significant predictor of EBV status before and after controlling for deprivation.

In conclusion, the results of this population-based study provide the first evidence of a strong association between EBV-positive HD and South Asian children from the United Kingdom. This was still significant after adjusting for deprivation. We have also demonstrated an association between material deprivation and EBV-positivity and this trend was still evident after adjusting for ethnicity, since the likelihood of EBV-positive disease was still increased in the most deprived patients.

### ACKNOWLEDGEMENTS

We wish to thank Dr F Raafat and Dr P Ramani, consultant histopathologists at Birmingham Children's Hospital, for their contributions to the pathology review. We also wish to thank Derek Lowe for his help with the preliminary analyses and Amanda Dutton for her help on the use of Townsend scores.

Research grant support: PGM is supported by a West Midlands NHS Executive LORS Award and the West Midlands Regional Children's Tumour Registry had financial support from the Leukaemia Research Fund and the Special Trustees of the former United Birmingham Hospitals Trust Funds.

### REFERENCES

Alexander FE, Ricketts TJ, McKinney PA and Cartwright RA (1991) Community lifestyle characteristics and incidence of Hodgkin's disease in young people. *Int J Cancer* **48**: 10–14

Ambinder RF, Browning PJ, Lorenzana I, Leventhal BG, Cosenza H, Mann RB, MacMahon EME, Medina R, Cardona V, Grufferman S, Olshan A, Levin A, Petersen EA, Blattner W and Levine PH (1993) Epstein-Barr virus and childhood Hodgkin's disease in Honduras and the United States. *Blood* **81**: 462–467

Andriko JAW, Aguilera NS, Nandedkar MA and Abbondanzo SL (1997) Childhood Hodgkin's disease in the United States: An analysis of histologic subtypes and association with Epstein-Barr virus. *Modern Pathol* **10**: 366–371

Armstrong AA, Alexander FE, Pinto Paes R, Morad NA, Gallagher A, Krajewski AS, Jones DB, Angus B, Adams J, Cartwright RA, Onions DE and Jarrett RF (1993) Association of Epstein-Barr virus with pediatric Hodgkin's disease. *Am J Pathol* **142**: 1683–1688

Armstrong AA, Alexander FE, Cartwright R, Angus B, Krajewski AS, Wright DH, Brown I, Lee F, Kane E and Jarrett RF (1998) Epstein-Barr virus and Hodgkin's disease: further evidence for the three disease hypothesis. *Leukemia* **12**: 1272–1276

Flavell K, Constandinou C, Lowe D, Scott K, Newey C, Evans D, Dutton A, Simmons S, Smith R, Crocker J, Young LS and Murray P (1999) Effect of material deprivation on Epstein-Barr virus infection in Hodgkin's disease in the West Midlands. *Brit J Cancer* **80**: 604–608

Glaser SL, Lin RJ, Stewart SL, Ambinder RF, Jarrett RF, Brousset P, Pallesen G, Gulley ML, Khan G, O'Grady J, Hummel M, Preciado MV, Knecht H, Chan JK and Claviez A (1997) Epstein-Barr virus-associated Hodgkin's disease: epidemiologic characteristics in international data. *Int J Cancer* **70**: 375–382

Grässer FA, Murray PG, Kremmer E, Klein K, Remberger K, Feiden W, Reynolds G, Niedobitek G, Young LS and Mueller-Lantzsch N (1994) Monoclonal Antibodies Directed Against the Epstein-Barr Virus-Encoded Nuclear Antigen 1 (EBNA 1): Immunohistologic Detection of EBNA 1 in the Malignant Cells of Hodgkin's Disease. *Blood* **84**: 3792–3798

© 2001 Cancer Research Campaign 

Gutensohn N and Cole P (1980) Epidemiology of Hodgkin's disease. *Semin Oncol* **7**: 92–102
Gutensohn N and Cole P (1981) Childhood social environment and Hodgkin's disease. *N Engl J Med* **304**: 135–140
Harris NL, Jaffe ES, Stein H, Banks PM, Chan JKC, Cleary ML, Delsol G, De Wolf-Peeters C, Falini B, Gatter KC, Grogan TM, Isaacson PG, Knowles DM, Mason DY, Muller-Hermelink H, Pileri SA, Piris NA, Ralfkiaer E and Warnke RA (1994) A Revised European-American Classification of Lymphoid Neoplasm: A proposal from the International Lymphoma Study Group. *Blood* **84**: 1361–1392
Jarrett AF, Armstrong AA and Alexander E (1996) Epidemiology of EBV and Hodgkin's lymphoma. *Ann Oncol* **7** (suppl 4): S5–S10
Lukes RJ, Carver LF, Hall TC, Rappaport H and Rubin P (1966) Report of the nomenclature committee in symposium: Obstacles to the control of Hodgkin's disease. *Cancer Res* **26**: 1311
Morris R and Carstairs V (1991) Which deprivation? A comparison of selected deprivation indexes. *J Public Health Med* **13**: 318–326
Murray PG, Swinnen LJ, Constandinou CM, Pyle JM, Carr TJ, Hardwick JM and Ambinder RF (1996) BCL-2 but not its Epstein-Barr virus-encoded homologue, BHRF1, is commonly expressed in posttransplantation lymphoproliferative disorders. *Blood* **87**: 706–711
Niedobitek G, Kremmer E, Herbst H, Whitehead L, Dawson CW, Niedobitek E, von Ostau C, Rooney N, Grässer FA and Young LS (1997) Immunohistochemical Detection of the Epstein-Barr Virus-Encoded Latent Membrane Protein 2A in Hodgkin's Disease and Infectious Mononucleosis. *Blood* **90**: 1664–1672
Office of Population Censuses and Surveys (1991) Standard occupational classification volume 3. London: HMSO
Pallesen G, Hamilton-Dutoit SJ, Rowe M and Young LS (1991) Expression of Epstein-Barr virus latent gene products in tumour cells of Hodgkin's disease. *Lancet* **337**: 320–322
Peh SC, Looi LM and Pallesen G (1997) Epstein-Barr virus (EBV) and Hodgkin's disease in a multi-ethnic population in Malaysia. *Histopathology* **30**: 227–233
Powell JE, Parkes SE, Cameron AH and Mann JR (1994) Is the risk of cancer increased in Asians living in the UK? *Arch Dis Child* **71**: 398–403
Preciado MV, De Matteo E, Diez B, Menárguez J and Grinstein S (1995) Presence of Epstein-Barr virus and strain type assignment in Argentine childhood Hodgkin's disease. *Blood* **86**: 3922–3929
Razzouk BI, Gan YJ, Mendonça C, Jenkins JJ, Liu Q, Hudson M, Sixbey JW and Ribeiro RC (1997) Epstein-Barr virus in pediatric Hodgkin Disease: Age and Histiotype are more predictive than geographic region. *Med Pediatr Oncol* **28**: 248–254
Stiller CA, McKinney PA, Bunch KJ, Bailey CC and Lewis IJ (1991) Childhood cancer and ethnic group in Britain: a United Kingdom Children's Cancer Study Group (UK CCSG) Study. *Brit J Cancer* **64**: 543–548
Townsend P, Phillimore P and Beattie A (1988) Health and deprivation, inequality and the North. Croom Helm, London
von Wasielewski R, Werner M, Fischer R, Hansmann M-L, Hübner K, Hasenclever D, Franklin J, Sextro M, Diehl V and Georgii A (1997) Lymphocyte-predominant Hodgkin's disease: An immunohistochemical analysis of 208 reviewed Hodgkin's disease cases from the German Hodgkin study group. *Am J Pathol* **150**: 793–803
Weinreb M, Day PJR, Murray PG, Raafat F, Crocker J, Parkes SE, Coad NAG, Jones JT and Mann JR (1992) Epstein-Barr virus (EBV) and Hodgkin's disease in children: Incidence of EBV latent membrane protein in malignant cells. *J Pathol* **168**: 365–369
Weiss LM, Movahed LA, Warnke RA and Sklar J (1989) Detection of Epstein-Barr viral genomes in Reed-Sternberg cells of Hodgkin's disease. *New Engl J Med* **320**: 502–506
Wu TC, Mann RB, Charache P, Hayward SD, Stall S, Lambe BC and Ambinder RF (1990) Detection of EBV gene expression in Reed-Sternberg cells of Hodgkin's disease. *Int J Cancer* **46**: 801–804

 *© 2001 Cancer Research Campaign*
